# Intuition, empathy, and intellectual humility in psychotherapy. A philosophical perspective

**DOI:** 10.3389/fpsyg.2025.1590481

**Published:** 2025-05-20

**Authors:** Eugenia Stefanello

**Affiliations:** Department of Philosophy, Sociology, Education, and Applied Psychology (FISPPA), University of Padua, Padua, Italy

**Keywords:** intuition, empathy, intellectual humility, psychotherapy, accuracy, doctor-patient relationship, virtue epistemology, clinical judgment

## Abstract

Intuition is often considered a crucial tool in psychotherapy, especially in guiding the therapist's clinical strategy. However, the specifically epistemic question of how to guarantee its accuracy deserves to be better explored. Drawing on Bohart's account of intuition in psychotherapy, I will first distinguish intuition from the closely related but distinct phenomenon of empathy, and then argue that the epistemic role of intuition in psychotherapy seems to be that of a salience detector. Second, I will demonstrate why intuition must be accurate to be therapeutically effective, thereby avoiding potential related epistemic and ethical pitfalls. Finally, I will conclude by suggesting that Bohart's proposed reflective feedback loop process appears insufficient to achieve this result, and that the epistemic virtue of intellectual humility seems a prerequisite for initiating and successfully carrying out this process.

## 1 Introduction

Intuition evokes something mysterious, especially when it comes to psychotherapy. While the idea that the therapist relies on intuition to guide clinical judgments and thus choose the therapeutic path may raise concerns about its legitimacy (Gaudiano et al., 2011), there is a growing body of literature arguing for the inclusion of intuition in psychotherapeutic practice, considering it “an important tool for psychotherapy and even essential for the encounter with the patient when the specificity of his or her character structure eludes theoretical models” (Welling, 2005, p. 20; Stickle and Arnd-Caddigan, 2019; Charles, 2004). A particularly interesting epistemic question regarding the role of intuition in psychotherapy is how to ensure its *accuracy*, especially given how relevant it seems to be to the therapeutic relationship in general and to clinical judgments in particular. This paper will attempt to explore this problem from a specifically philosophical perspective, while also taking into account empirical evidence from neuro- and cognitive psychology.

To explore this issue further, I will first examine a specific account of intuition in psychotherapy, namely Bohart's perspective (Section 2). His proposal is particularly intriguing because it grounds the argument for the role of intuition in a solid conceptual foundation while also providing a paradigmatic case study that I will discuss in detailed. I will then attempt to clarify a potential source of confusion: intuition is distinct from, though complementary to, empathy (Section 2.1). This will allow me to better explain the specific epistemic contribution of intuition compared to empathy in the psychotherapeutic setting, i.e., as a salience detector (Section 2.2). I will then discuss the central issue of accuracy by showing why the accuracy of intuitive insights is crucial for effective therapy and for avoiding negative consequences, both from an epistemic and an ethical perspective. Because of its importance, I will question whether Bohart's solution to this problem, which I will call “the feedback solution”, is sufficient to achieve the goal of accurate intuition (Section 3). Finally, I will argue that for the feedback loop to be effective in ensuring the accuracy of intuition, the epistemic virtue of Intellectual Humility (IH), especially as characterized by Alessandra Tanesini, can be a powerful ally.

## 2 Intuition in psychotherapy: Bohart's account

One of the most insightful discussions of the role of intuition in psychotherapy is found in Bohart's (1999) article *Intuition and Creativity in Psychotherapy*, where he argues for the pervasive and critical role of intuition in our cognitive process in general and in the therapeutic relationship in particular, both of which he describes as inherently creative (Bohart, 1999). More specifically, he argues that all of our cognitive acts are creative in themselves and, crucially, that intuition is the starting point of any cognitive-creative process as they all begin with having a perceptual insight or “felt sense” about something in a nonverbal and non-conceptual way. In this respect, cognition is analogous to aesthetic perception, since at the most basic level human beings know the world aesthetically, recognizing patterns, rhythms, flows, etc., before they consciously conceptualize it. Intuition is precisely responsible for identifying these qualities and grasping their epistemic content. Thus, the first step in the cognitive-creative process is this intuitive, bodily and experiential grasp of the world, of others, and of ourselves.

The epistemic content that intuition gathers constitutes a specific kind of knowledge, namely tacit knowledge, which is often discussed in the context of expertise. Polanyi famously argued that since “we can know more than we can tell,” there is a kind of knowledge that we possess but are unable to fully articulate and thus communicate to others (Polanyi, 2009). For this reason, tacit knowledge can be developed through experience over time rather than through step-by-step guidelines or manualized instructions (Ribeiro, 2013). Thus, in Bohart's view, intuition seems to be one of the most effective tools available to the knower to gain this kind of knowledge, as it allows access to a rich set of information in a perceptual, bodily, and experiential way before we can articulate it verbally (Bohart, 1999). Intuition is also spontaneous, below the level of consciousness, and instantaneous, characteristics that are both epistemically compatible with the nonverbal and non-conceptual nature of tacit knowledge, but also particularly useful in the context of practicing a profession or activity that requires making multiple decisions or judgments in a short period of time.

In fact, according to Bohart, intuition and tacit knowledge are particularly important in psychotherapy. Therapists must use intuition to grasp what is happening in the flow of the therapeutic interaction and relationship in order to make their clinical judgments. In particular, therapists rely on intuitive recognition and sensing of patterns, rhythms, and flows in the patient's experience, in the therapeutic relationship, and in their own inner experience to guide their interventions and moment-to-moment judgments in deciding how to deal with a particular condition and with patients in general (Bohart, 1999).

The epistemic and therapeutic power of intuition in psychotherapy seems to emerge powerfully from a case study described by Bohart in which he creatively used his intuition to change his approach to a patient named David who was depressed because of his almost complete lack of significant relationships (e.g., friends, coworkers, romantic partners) in a way that opened up new therapeutic possibilities (Bohart, 1999, p. 305–309). Initially, Bohart used a traditional patient-centered approach with David, focusing solely on establishing an empathic relationship. However, he was dissatisfied because this approach was not working with the patient, who was extremely guarded and continued to over-intellectualize every aspect of his life and their therapeutic interactions. Suddenly, however, Bohart had an intuition that made him realize that while staying true to the patient-centered principles of being empathic, he could also radically change the way he interacted with David and hopefully try to initiate a change in his cognitive and affective approach to his therapy and to life in general.

The intuition was to realize that by engaging David in lively arguments and debates during sessions on a wide range of topics not limited to his personal life and problems, in addition to their empathic relationship, Bohart could open up a new dynamic in which David could become more emotionally expressive and aware of his patterns of thought and behavior in a way that the previous empathic relationship had not allowed. In order to implement his clinical judgment and plan, Bohart had to change his attitude by openly arguing and disagreeing with David. Bohart's intuition not only sparked the realization that open discussion and disagreement might have been beneficial for David, but also allowed Bohart to establish some intuitive principles to follow during the process, such as using first-person statements, admitting when one is wrong, and not focusing on “winning” the argument for the sake of feeling intellectually superior, in order to show David a different way of approaching discussions than he was accustomed to doing.

Bohart described acting on this intuitive hunch in a spontaneous, improvisational way, without a predetermined plan or manual to follow, creatively configuring the therapy process in the moment based on his intuitive judgment of what this particular patient needed. The patient also creatively engaged with this radical shift in therapy format which, over time, allowed him to spontaneously revise some of his rigid beliefs and behaviors. In particular, David began to realize how emotionally invested he was in trying to win them, contrary to his belief that only logical and rational thinking mattered. At the same time, by experiencing arguments in which he sometimes won and sometimes lost, David became less set on winning as a way to boost his self-esteem and more aware of how his behavior could alienate people, which also had a positive impact on his romantic relationships with women and his overall wellbeing.

David's case allows us to discuss two crucial aspects of the role of intuition in psychotherapy as presented in Bohart's account. First, intuition is understood here as distinct from empathy in shaping the therapeutic encounter. This is far from obvious, since these are two phenomena that could be seen as overlapping. After all, even in their commonsense characterizations, they both refer to something that gives us a kind of shortcut or quick insight into a situation or another person, which of course seems particularly useful in the therapeutic context. The temptation to make these two concepts coincide in the therapeutic context may also be due to the fact that they both seem to be aimed at helping the therapist to achieve a tailor-made approach to each individual patient by grasping his or her specific conditions and needs (for a review see Gibbons, 2011). In what follows, however, I will try to show why empathy—understood not only as Bohart does, but also in several other possible definitions—should be conceptually distinct from intuition. Second, David's case also provides an opportunity to try to fill what I see as a gap in Bohart's account, namely the lack of a more detailed characterization of the specific epistemic role and contribution that intuition is supposed to play in clinical judgment and in the therapeutic relationship as a whole.

### 2.1 The difference between intuition and empathy

Bohart explicitly distinguishes empathy, as understood in patient-centered therapy, from intuition, while considering it complementary. According to this therapeutic approach, pioneered by Carl Rogers and adopted by Bohart himself, the therapist aims to create an accepting and positive atmosphere by continually attempting to verbally and affectively reflect the patient's inner experience so that the patient feels understood (Rogers, 1975; Bohart and Greenberg, 1997). The core insight is that by providing this empathic framework, the therapist helps the patient explore and process their thoughts and feelings in a safe, non-judgmental space (Elliott et al., 2023). For this reason, the therapist usually refrains from offering direct advice or interpretation of the patient's experience and instead uses reflective listening responses such as, “It sounds like you are really frustrated about this situation” or “I can see that this person's behavior is making you really sad”.

Bohart himself claims that his initial “goal [with David] was to provide an empathic relationship, and that was all. My goal—he concludes—was to be a certain kind of person with him, to provide a healing environment” (Bohart, 1999, p. 306). While even responding empathically to a particular patient requires creativity, as the therapist “still needs to construct empathy responses inventively on a moment-to-moment basis”, intuition seems to provide a much stronger epistemic contribution that is then used to inform the therapist's response and therapeutic intervention (Bohart, 1999, p. 301). While empathy is certainly necessary for the success of the therapeutic interaction, it does not appear to be the source of the intuitive insights that are critical for directly shaping therapeutic judgment and intervention.

However, one could say that the Rogerian definition of empathy adopted by Bohart is hardly the only one available in the literature, both in psychotherapy and in social cognition more broadly (Watson, 2016). In fact, empathy is often understood as making a much stronger epistemic contribution to the therapeutic relationship than what the patient-centered approach proposes. In this context, empathy should be able to provide the therapist with access to the patient's interiority by imaginatively adopting the patient's perspective (Spaulding, 2017; Gehlbach and Mu, 2023; Staemmler, 2012). By empathically taking the patient's perspective, therapists are supposed to achieve two important results. First, they are supposed to gain new information about the patient's condition that would not otherwise be available; and second, they are supposed to gain a deeper understanding of the information that the patient explicitly discloses. In this view, empathy does more than simply reflect back and respond to the meanings expressed by the patient, because it allows the therapist to make substantive epistemic gains about the patient's internal states.

While defining empathy as perspective-taking may seem to blur the line with intuition, I would argue that they remain distinct concepts. Although their epistemic outcomes may partially overlap, since both aim to understand the patient's specific condition in order to better address his or her needs, they differ greatly in their underlying processes. In contrast to intuition, empathy as perspective-taking is a fully conscious and explicit process that can be described, developed, and, if necessary, taught through step-by-step instructions, as evidenced by the fact that perspective-taking is considered by some to be a valuable strategy for mitigating some biased attitudes toward people we identify as members of out-groups (Lamm et al., 2007). Thus, whereas intuition operates on a tacit, unconscious level, cognitive empathy involves the conscious cognitive effort of deliberately taking another's perspective (Decety and Jackson, 2004; Goldman, 2011).

At this point, one might concede that the more cognitive aspect of empathy, i.e., perspective-taking, is distinct from intuition, but argue that the affective side of the empathic phenomenon might render this distinction superfluous. Again, I remain unconvinced. First, if we define affective empathy as affective sharing, i.e., being in the same emotional state as the other person, we are still talking about a conscious and deliberate, albeit affectively charged, process (Maibom, 2017; Guo, 2017). When the therapist empathizes with her patient's emotional state, she does so knowing that the source of her emotion is her patient and that she can initiate or interrupt this process at any moment. The intuitive process, on the other hand, unfolds on a completely different level, which, moreover, does not imply the need to personally experience the patient's emotional state in order to grasp it, as affective empathy does.

Second, even if we consider the related phenomenon of emotional contagion, which consists of feeling uncontrollably and spontaneously what the other person is feeling without being aware of the origin of the emotion (e.g., feeling cheerful by being around cheerful people), the distinction with intuition seems to persist. This is because, while both are unconscious, only intuition seems capable of providing valuable epistemic insight into the other's affective state. Precisely because in an instance of emotional contagion the subject does not know why she is experiencing that emotion, she is unable to access information that is valuable in guiding her response to the situation, which makes emotional contagion not only different from intuition, but also extremely less valuable—and potentially problematic—especially in the context of psychotherapy (Elfenbein, 2014; Hatfield et al., 2009).

There is a final characterization of the empathic phenomenon that threatens to collapse the distinction with intuition: the phenomenological account of empathy. In this approach, empathy is seen as a direct quasi-perceptual experience of the other's inner states through his or her bodily gestures and verbal manifestations, which can be further explored by following the other's intentional objects in order to better grasp the motivational context (e.g., the reason why) of that behavior (Stein, 1917; Jardine and Szanto, 2017). It could be argued that empathy, so understood, seems to function similarly to intuition, directly grasping the verbal and nonverbal cues as well as the patient's behavior.

While I agree that the directness of empathy as characterized by the phenomenological approach is consistent with some aspects of intuition, I would not consider them to be the same phenomenon. There are two reasons for this. First, phenomenological empathy consists of both a spontaneous and “always active” moment—a quasi-perceptual acquaintance with the manifestations of the other's internal states (e.g., I see the other's shame in her blush)—and a more conscious, effortful, and discretionary moment of following the other's intentionality, which allows the empathizer to grasp the motivational structure in which his or her internal states are located (e.g., I understand that the other is ashamed because she failed her exam) (Jardine, 2015; Zahavi, 2014). Thus, while the first “moment” of the empathic process shares some features with intuition, its second “moment” seems to have an epistemically distinct status from intuition, as well as from its spontaneous and automatic genesis.

Similarly, the difference between the first “moment” of phenomenological empathy and intuition becomes clear when we look at the inherent “problem-solving quality” of intuition, which is absent from phenomenological empathy in either its first or second “moment”. Whereas intuition is directed toward creatively working out a possible solution to a problem, challenge, or change in the subject, empathy, according to Phenomenology, consists only of a modality in which we exist with and understand some aspects of the consciousness of others as well as shed some light on the world we share with them. Thus, in the phenomenological perspective, the empathically acquired information about the other has no other goal than to gain a basic understanding of the other's inner states. Intuition, on the other hand, seems to have a broader and more specific epistemic role when it comes to psychotherapy and cognitive processes in general. Clarifying this role is what I will try to do next.

### 2.2 Intuition as salience detector in psychotherapy

While the overarching role of intuition is to obtain the best therapeutic plan possible, its specific epistemic contribution needs to be better articulated. From what it emerges from Bohart's discussion, intuition seems to be responsible not only for picking up implicit cues or tacit information from both patient and therapist, but also for sorting out what is salient from what is not. It seems to me that only by attributing to intuition the additional ability to select the relevant information from the plethora of information available during a therapeutic interaction can we explain how it can provide the therapist with valuable insights about the patient and, most importantly, guide the therapist in resolving the potential problems that arise during the process.

For this reason, I suggest that intuition should be characterized as a *salience detector* aimed at solving issues, rather than a mere information collector. Because of the sheer amount of information generated in therapy sessions, where uncovering implicit meanings or thoughts and emotional states is crucial, the ability to identify what is most relevant seems to be the crucial feature of intuition as well as what makes it different from empathy variously defined.

Thus, in the therapeutic context, intuition seems to allow the therapist to focus on and identify which of the patient's cues and observations and the therapist's own inner reactions are most meaningful. It is this salience detection function that seems to enable intuition to generate insights that can creatively address problems and guide the therapist's clinical judgments: rather than simply collecting all the information available, intuition acts as a filter. Without this ability to identify what is most salient, the therapist could be overwhelmed by the volume of subtle but potentially relevant information that arises in each session.

The claim that therapists need some tools to sort through all the information patients' provide them with is rather uncontroversial. For instance, Fowers and colleagues argue that “the sheer amount of incoming information means that psychotherapy requires a constant focus on separating the trivial from the important” and that this process cannot be accomplished by technique or theory because they are too “abstracted, generalized, and schematized to apply to a wide range of situations (Fowers et al., 2022). Therefore, they cannot specify the appropriate responses across radically diverse and specific clinical situations” (Fowers et al., 2022, p. 701). Contrary to my claim, however, Fowers and colleagues suggest that it is the therapist's practical wisdom that is responsible for signaling which aspects of the flow of information are most relevant.

While I agree with them that practical wisdom is certainly a good virtue for the therapist to possess, and that it can contribute to some extent to the signaling of the most salient information (Darnell et al., 2019), I have some doubts that this particular trait is capable of sustaining the “epistemic pace” that this process requires in the therapeutic context: I am not sure that a conscious and fully explicit trait like practical wisdom is capable of guiding a much faster and more pervasive process like salience detection. In contrast, intuition seems up to the task, given its speed and implicitness, which also make it less cognitively demanding than practical wisdom. Interestingly, Fowers and colleagues seem to recognize this as well, noting that “of course, this perceptual process (…) is not an entirely explicit and conscious process. There is always some degree of guidance form intuition and hunches, but recognizing salience is very early and essential part of therapeutic understandings” (Fowers et al., 2022, p. 709).

Thus, intuition seems to be a good candidate to be the first and most basic tool—though potentially not the only one—that therapists have for recognizing what information is most salient in their encounters with patients. The idea that the role of intuition is to detect salient clues among the many provided by patients is also shared by Welling, who argues in what he calls the “related object phase” that the therapist is able to identify elements or objects (e.g., the patient's remarks, gestures, themes) as relevant through the therapist's intuitive feelings (Welling, 2005, p. 36). Some examples include the therapist feeling that a client's hand movement or certain details “stand out” as meaningful, or noticing an incongruity between the client's words and actions. Most notably, in his famous *Notes on memory and desire* Bion argued that the psychotherapist should resist relying on memory of past sessions or desires for particular outcomes, as these interfere with genuine observation and judgment (Bion, 1988). Crucially, the psychotherapist should engage in the largely unconscious work of intuiting the psychic reality of the present moment by becoming one with the unconscious psychic truth of the patient (Ogden, 2015). Thus, in order for the therapist to be able to discern the relevant and true evolution of the patient's experiences, as well as the therapeutic interaction itself, the therapist must rely heavily, if not exclusively, on his or her intuition.

In David's case, it was this salience-detecting function of intuition that seemed to allow Bohart to suddenly choose to engage in lively debates with the patient. First, the immediacy and reflectiveness with which this process and its outcome took shape in the therapist's mind seems to point to intuition rather than to practical wisdom.^1^ Moreover, if the role of intuition had been simply to assess what David was thinking or feeling by picking up on all the implicit cues between them, Bohart might have been more aware but might not have found the solution to her therapeutic impasse.

For example, the fact that Bohart did not directly address David's difficulties in forming sexual relationships with women seems to have stemmed from an intuitive understanding that it would not have been productive to directly challenge David's strict religious beliefs as the cause of his interpersonal problems. Thus, Bohart's creative intuition was not only that “the interaction itself has to be the therapy” but also that David's religious beliefs were not as salient a factor in this particular therapeutic strategy as they might initially have seemed (Bohart, 1999, p. 307).^2^ To be clear, his strict religious beliefs were salient to the overall clinical picture, but not so salient when it came to this particular intuitive and creative therapeutic judgment and consequent intervention.

If my claim that intuition's specific epistemic role in psychotherapy is to act as a salience detector is plausible, then a crucial question arises: how do we ensure the accuracy of intuition in capturing and filtering information? In other words, how do we ensure that intuition accurately identifies the most relevant information and insights? In the next section, I will discuss Bohart's preliminary answer to this problem. However, his proposed solution seems to only partially address this accuracy concern. Therefore, in the final section, I will propose the epistemic virtue of Intellectual Humility (IH) as a complementary solution for promoting accurate intuitions and resulting problem-solving strategies in the therapeutic context.

## 3 Intuition, the problem of accuracy, and the feedback loop solution

Intuition as salience detector is only half of the story. As Bohart argued, this kind of intuition is then attempted to be articulated through words, symbols, metaphors, etc., and, in the specific context of psychotherapy, through questions, interpretations or interventions (Bohart, 1999, p. 293–298). Most importantly, it must go through a cyclical process of being checked against the original felt intuition, leading to its sharpening and revision. It must also be tested against external evidence, objections and contradictions, with further revision based on how well the original articulation fits with the responses of other subjects and the objective evidence of the world. This process of internal and external validation and verification is essentially a process of editing, continually refining the initial intuitive insight and its articulation.

This conceptual work is as crucial as the original non-verbal intuition that initiates the creative process. In Bohart's words, “there is a process of: sensing/perceiving → symbolizing; checking of the symbol against felt experience, leading to a sharpening of felt experience, leading to refined symbolizing, and so on, until one has a well-formed creative product” (Bohart, 1999, p. 298). Because of its iterative and circular nature, I propose to characterize this process in terms of a feedback loop.

Note that this process is intended to address two potential sources of inaccuracy: either the intuition itself could be flawed, or a sound intuition could be inaccurately translated into words and concepts, leading to an inaccurate result, i.e., inaccurate clinical judgment. For this reason, there seems to be a two-step verification process at play. First, the intuition itself is tested against internal and external evidence from the subjects and the world, and second, the conceptual articulation of the intuition is tested against felt experience and external evidence, which in turn can trigger further revision of both the symbolization and the intuition itself in a potentially infinite loop.

Although Bohart does not frame his proposal in this way, it seems quite consistent with the Dual-Process Theory (DPT) approach. According to a large body of empirical evidence, two types of thinking can be distinguished: intuitive and reflective. The former is usually characterized as fast, unconscious, automatic, and associative, and the latter as slow, conscious, controlled, and rule-based (Evans, 2008). Typically, Type 1 processes represent the “default mode,” *qua* unconscious, in which we largely make decisions, while Type 2 processes are used more selectively (Kahneman, 2013; Gawronski and Creighton, 2013; Neys, 2018). Because of their automatic and less effortful nature, Type 1 processes are thought to meet the efficiency demands of our everyday lives, whereas Type 2 processes are involved in decision-making tasks that require more flexibility, such as those that involve considering the future implications of the choice.

The interplay between these two processes is particularly relevant in social cognition and in complex decision-making situations, of which psychotherapeutic interaction is a clear example. Consistent with Bohart's feedback loop, the DPT argues that “reasoning and decision making sometimes require both (a) an override of default intuition and (b) its replacement by effective Type 2, reflective reasoning” (Evans and Stanovich, 2013, p. 236). It follows that, according to (this version of) DPT, “most of our behavior is controlled by Type I processes running in the background” and that, triggered by various factors such as obstacles, novelty, or, importantly, “metacognitive feelings of rightness in the initial intuition”, System 2 processes are prompted to intervene (Evans and Stanovich, 2013, p. 236).

This idea that intuitions may initially arise with a sense of rightness but are not automatically correct, and that the feedback loop must therefore always be active, is openly discussed by Bohart himself in the specific context of psychotherapy. Precisely because he was aware that his initial intuition in David's case might have been wrong, and that the therapeutic strategy based on it might not have worked effectively, he “was also continually monitoring to make sure that what [they] were doing seemed to be a productive process for [them] both” (Bohart, 1999, p. 308). Thus, as both Bohart's argument and neuroscientific and psychological evidence show, the process of editing and revising intuitive insights, carried out by what DPT theory calls System 2, is a common and crucial feature of our cognitive processes, including clinical judgment formation in psychotherapy.

An important implication seems to follow from this. At least from a conceptual standpoint, there seems to be a correlation between the accuracy of the epistemic content provided by intuition, its translation into practice through various modes of conceptualization, and its therapeutic outcomes. More specifically, accuracy seems to be a necessary, though not sufficient, condition for achieving a positive therapeutic outcome. The core idea is that for intuition to be therapeutically productive, both the information intuitively grasped and the criteria used to determine its salience seem to need to be consistent with the actual conditions of the patient, the therapist, and external evidence.

This conceptual correlation between accuracy of intuition and therapeutic success is quite clear in David's case. One of the reasons that Bohart's intuitive approach worked with David was that the insights it provided accurately captured key aspects of David's situation while leaving aside others that were not as relevant to the specific therapeutic intervention. In addition, Bohart's way of setting up and leading the discussions (recall all the rules he sets for himself) also had to reflect their behaviors, needs, and overall situation.^3^

Another way to show this conceptual correlation is to consider what happens when the epistemic content of intuitive insights is inaccurate and the therapist is unable to correct it. In particular, there seem to be two potential risks for the patient and for the clinical encounter as a whole. On the one hand, there is the risk of usurping the patient's epistemic authority by mischaracterizing his or her specific needs and inner experience due to the therapist's inaccurate intuitions (Zagzebski, 2012). Since both the intuitive process and much of the patient's lived experience, emotional states, and meanings are already difficult to articulate because they are mostly below the level of consciousness, the likelihood of error increases dramatically, which, combined with the high stakes involved in the therapeutic setting, requires the therapist to be especially careful not to over-impose his or her own inaccurate intuitive insights on the patient. If the therapist is unaware of his or her error by not being receptive to the patient's feedback, he or she may not only choose a therapeutic strategy that is likely to be ineffective, but may also significantly hinder his or her own ability to truly understand the specific and individual condition of that patient.

On the other hand, this epistemic pitfall can lead to an ethically problematic lack of respect for the patient's agency in determining the course of his or her own therapeutic process, which should be informed by the therapist's expertise, of course, but also shaped by the patient's own specific goals and needs (Fisher and Oransky, 2008). In other words, each patient should be able to both implicitly and explicitly influence, correct, or modify the therapist's judgments, that is, the therapist's understanding of the patient's lived experience and related therapeutic choices. Thus, inaccuracy seems to be conceptually negatively correlated with therapeutic success because it risks undermining both the epistemic goal of achieving accurate understanding and the ethical requirement of respecting the patient's self-determination and autonomy over his or her own condition and therapeutic plan, which is crucial in any clinical encounter.

As noted above, the antidote to these potential risks of inaccurate intuition proposed by Bohart is the feedback loop process. While I agree that this iterative process seems capable of correcting and refining intuitive insights as well as informing the role of intuition in recognizing salient therapeutic cues, it does not seem sufficient on its own to fully achieve the level of accuracy required in the therapeutic relationship. This is not because the process itself is ineffective. Ongoing revision, editing, and correction of intuitive insights with multiple epistemic sources through reflection is the only way to attempt to address, though never definitively, the gaps that inevitably affect our cognitive processes. Rather, there seems to be a specific epistemic disposition, Intellectual Humility (IH), that would be not only useful but necessary for the therapist to both recognize the need to initiate this process and then to incorporate its results. In the next section, I will try to show precisely why I believe that IH is a useful, if not a necessary, prerequisite for intuition to be accurate and thus effective in the therapeutic relationship. Without this disposition, even the feedback loop may be insufficient to verify the accuracy of intuitive insights and judgments and thus avoid the potential epistemic and ethical violations.

## 4 Intellectual humility as the precondition for accurate intuition in psychotherapy

To see why IH might contribute to the accuracy of the intuitive process, it is necessary to define it precisely. Broadly speaking, there are three major approaches to its definition (Alfano et al., 2021). First, it consists of being ignorant of one's own merits (Driver, 1989). Second, it coincides with being aware of one's intellectual limitations or the ability to modulate confidence in one's own beliefs (Whitcomb et al., 2017; Kidd, 2015). Third, it consists of a low level of self-focus in general, coupled with a low concern for one's social status and self-worth, as well as a high level of other-focus (Roberts and Wood, 2007). Thus, IH is usually understood as either a virtue of ignorance about one's own cognitive achievements, a virtue of accuracy about one's own cognitive abilities, or a virtue of appropriate concern (i.e., low for the self and high for others) regarding one's intellectual pursuits.

However, as Alessandra Tanesini rightly points out (Tanesini, 2018), these accounts all focus on only one aspect of IH, thereby missing what she considers to be the dual nature of this virtue, namely, as composed by modesty and self-acceptance. According to her, modesty “concerns the motives or causes for a person's positive stance toward her epistemic successes” (Tanesini, 2018, p. 403). If a person is modest means that she considers her cognitive achievements for their actual epistemic value and not because of their positive impact on her social status or self-esteem (Tanesini, 2018, p. 403).

Concurrently, Tanesini defines the self-acceptance dimension of IH as the quality of being “accepting of limitations that she attributes to herself” (Tanesini, 2018, p. 405). One of the most relevant ways to display this quality is to openly, willingly, and autonomously recognize one's own shortcomings, flawed intellectual outcomes, or weaknesses. This recognition consequently leads the person to be open to “fair criticisms with equanimity [without being] resentful of them” (Tanesini, 2018, p. 405). Importantly, while being psychologically unified, i.e., they tend to manifest simultaneously, modesty and self-acceptance are two conceptually distinct qualities of the knower.

Crucially for my purposes, the self-acceptance dimension of IH as characterized by Tanesini seems to provide an ideal conceptual framework for demonstrating why this virtuous trait seems to be, if not a necessary precondition, at least useful for the accuracy of intuitive insights and related judgments. There are two reasons for this: its epistemic goal and its motivational role. First, the epistemic goal. According to Tanesini, IH consists of having positive attitudes toward aspects of one's cognitive makeup that promote epistemic goods such as accurate understanding. The humble person values attitudes that indicate a desire for truth, knowledge, and understanding (Tanesini, 2018, p. 412). Thus, the specific goal underlying self-acceptance is the attainment of accurate understanding by being positively oriented toward those aspects of one's cognitive capacities that contribute to the attainment of this epistemically virtuous end, and negatively oriented toward those aspects that impede it.

Second, the motivational role. Self-acceptance is characterized not merely as a form of knowledge, but as a “concern, […] a way of caring that one has limitations because of their effects on the pursuit of various epistemic goods” (Tanesini, 2018, p. 405). Because of this concern about the consequences of one's intellectual limitations, the humble person is motivated to directly address and correct these shortcomings. The affective nature of this concern/care actively motivates the knower to revise and thus improve their understanding.

It is this specific concern element, combined with its ultimate goal of achieving accurate understanding and thus actively correcting errors, that I believe is useful or even necessary to responsibly use the creative potential of intuition while mitigating its risks in a high-stakes domain such as psychotherapy. Specifically, the feedback loop process that should guarantee the accuracy of the intuitive insights, as well as the therapeutic actions based on those intuitions seems to require IH, especially its self-acceptance dimension, in order to be properly initiated and continuously maintained.

This is because concern and care for the implications of one's cognitive makeup for accurate understanding in general, and for one's intuitive insights in particular, seems to be necessary for the therapist to be motivated not only to initiate and maintain the feedback loop process regarding one's intuitions, but also to remain open, i.e., motivated, to actually correct those intuitions when the situation requires it. In other words, for intuitive insights to be as accurate as possible, the feedback loop process must always remain active, and the therapist must always be receptive to its results. This, in turn, can happen if the therapist maintains a self-accepting attitude toward his or her own epistemic limitations. Such an attitude motivates them to be alert to the need to correct and modify their previous intuitive insights as needed in order to ultimately achieve accurate understanding.

The positive correlation between accuracy and IH appears to be empirically supported, as IH has been shown to foster positive traits such as curiosity, careful consideration of evidence for or against one's own and others' claims, and an overall greater openness to testing and revising the validity of the information one encounters (Leary, 2022; Leary et al., 2017; Krumrei-Mancuso et al., 2020; Porter et al., 2022). Precisely because intellectually humble knowers are more attentive and open to the possibility that their own intuitions or thoughts in general might be wrong, “IH seems to enhance knowledge, understanding, and the quality of people's decisions” (Leary, 2022, p. 1402).

Additionally, the claim that IH is able to motivate the knower to achieve accurate understanding seems also corroborated by several empirical findings. In particular, intellectually humble people seem to simultaneously have higher motivation to perform certain cognitive tasks, such as problem solving, learning new things, and being open to challenging information, and lower motivation to achieve “cognitive closure”, which is, “the degree to which people are motivate to make decisions quickly and are reluctant to revisit decisions they have made” (Leary, 2022, p. 1403). Thus, the intellectually humble person appears to be more motivated to spend time and cognitive resources to understand a situation accurately, even if this means accepting a degree of uncertainty and the possibility of ongoing revision, than to arrive at a definitive, albeit incorrect, solution.

Importantly, because IH in general, and self-acceptance in particular, is an attitude, it does not always require conscious reflection on one's cognitive make-up. Rather, these evaluations can be “habitual and quasi-automatic” (Tanesini, 2018, p. 410). This is because mature individuals develop an overall impression of their intellectual strengths and weaknesses through continuous assessment of their cognitive characteristics and intellectual performance. Thus, while these evaluations are sometimes based on conscious reflection, they often arise from “lessons learnt unthinkingly” in the knower's past experiences (Tanesini, 2018, p. 410).

This characterization of IH as both habitual and quasi-automatic as well as reflective and deliberate seems to fit conceptually well with its role as a prerequisite for accurate intuition. On the one hand, the irreflective nature of self-acceptance, is consistent with the irreflective nature of intuition itself. On the other hand, its more reflective elements seem to be able to effectively dialogue with the System 2 processes involved in editing and revising intuition. This also seems to be supported by empirical evidence that IH has several psychological underpinnings (e.g., mental flexibility and analytical thinking) that are common to both systems (Zmigrod et al., 2019).

Let's revisit David's case one last time. In my view, only an intellectually humble therapist would have kept his intuition and related judgment open to revision if the patient had raised concerns or not responded positively. This is because in an intellectually humble knower, the epistemic motivation that drives the cognitive-intuitive process is to arrive at an accurate intuitive insight that is indispensable for pursuing the patient's best interests and the optimal therapeutic course. It is because Bohart cared about the epistemic good of arriving at an accurate intuitive understanding that he was both receptive to his own cognitive limitations and open to modifying his decision based on his intuition, which in turn was continually checked against patient feedback and corrected as needed.

The effectiveness of IH on the therapeutic relationship has recently been discussed by Davis and Cuthbert, who, after reviewing the empirical literature, argue that humility appears to be critical in facilitating a strong therapeutic relationship, which in turn produces positive patient outcomes (Davis and Cuthbert, 2016). In particular, they show how humility appears to underpin many other positive attitudes and skills, such as collaboration and alliance, that promote a productive psychotherapist-patient relationship. They also suggest some recommendations for therapists to cultivate this trait, such as seeking feedback, engaging in deliberate practice to improve effectiveness, and developing accurate self-assessments of their skills and competencies, which are entirely consistent with the characteristics of IH discussed above.

Similarly, Sandage and colleagues argue that “in the context of psychotherapy, humility represents the developmental progress of clinicians toward accurate understanding of one's skills and limitations as a healer” (Sandage et al., 2016, p. 305). Relevantly, they describe one of the expressions of humility in psychotherapy as “adopting a stance of *not knowing*,” which consists of an attitude that tolerates ambiguity and respects the patient's perspective, as he or she “may know better than the therapist in any given moment” (Sandage et al., 2016, p. 310)^4^. This awareness seems not only compatible with Tanesini's account of IH but also crucial for initiating and keeping open the feedback loop process discussed above. More generally, IH appears not only to prevent therapists from becoming overly attached to particular models or theories that may not fully capture the complexity of a patient's experience, but also to encourage therapists to actively seek further training and consultation when needed. For this reason, it may be beneficial to include IH in training programmes by discussing its conceptual features and strategies for cultivating it in clinical practice, as well as modeling it during the supervision phase.

To be clear, I am not suggesting that IH is the only epistemic virtue the therapist must possess in order to arrive at accurate intuitive insights and thus provide the best possible care. While it seems to be useful, if not a necessary condition, it is not sufficient, since other epistemic virtues, such as open-mindedness and honesty, are needed to achieve these goals. Again, this seems consistent with the account of IH proposed here, since one of the features of this virtue is the ability to promote and be promoted by other epistemic virtues (Tanesini, 2018, p. 408). This is because while the core attitudes and behaviors that constitute IH are primarily related to the self (being modest and self-accepting of one's cognitive traits), they can also be related to other attitudes and behaviors directed toward others, such as other epistemic virtues (Leary et al., 2017).

Finally, there are some limitations to consider. This discussion has focused primarily on the psychotherapist's intuitive insights and IH, examining the patient's reactions and lived experiences as one of the main sources of information in the feedback loop process. Further exploration might address the role of the patient's own intuition in the therapeutic process, and whether promoting IH might be a valuable therapeutic goal for patients themselves. Furthermore, while the interplay between intuition, empathy, and IH has been discussed in the context of individual therapy, it may be useful to examine these dynamics in other therapeutic settings, such as couples, family, or group therapy. Similarly, while this analysis deliberately did not refer to any specific therapeutic approach, since intuition and the intuitive habit are proposed as common elements across all therapeutic relationships, it could be valuable to further investigate how their roles might vary depending on the different therapeutic orientations.

## 5 Conclusion

My goal was to explore the critical role of intuition in psychotherapy and to argue for the need to integrate the intuitive process with IH. In Section 2, after briefly discussing Bohart's influential account of intuition in psychotherapy as the initial creative starting point that guides therapeutic interventions, I first distinguished intuition from empathy: intuition provides epistemically rich problem-solving insights, while empathy facilitates a connection with the patient. I then argued that by framing intuition as a salience detector, we may be able to better clarify the specific epistemic contribution it makes in the therapeutic setting. In Section 3, I attempted to show why the accuracy of intuitive insights and related judgments is crucial for effective therapy and for avoiding negative consequences, both from an epistemic and an ethical perspective. Given the importance of accuracy, I then questioned whether the feedback loop process proposed by Bohart is sufficient to guarantee it. This led me to suggest in the final section that IH, particularly its self-acceptance dimension, should be considered, if not a necessary condition, at least an extremely useful one, for ensuring the accuracy and effectiveness of intuition in therapy.

While more empirical research is needed to further examine the relationship between IH and intuition accuracy in the therapeutic setting, there appears to be a positive conceptual relationship between the two, which is also supported by several empirical findings. This also suggests that developing strategies to enhance both the critical revision of intuitive insights and the virtue of IH may lead to more effective and satisfying therapeutic relationships for both therapists and patients.

## Footnotes

^1^To reiterate, this does not exclude the possibility that practical wisdom may have played a role in guiding other aspects of the therapist's actions and decisions, while reserving the initial and fundamental function of salience detection and the consequent decision to change the therapeutic strategy to intuition.

^2^Evidence of this is the fact that after the shift to an argumentative approach, David decided to have a sexual relationship with a woman before marriage without discussing it beforehand with Bohart.

^3^Note, however, that it does not follow that accuracy of intuition is all that is needed for a successful therapeutic interaction. Other equally important elements must be present to achieve this result (e.g., a therapist who is warm, open, and always available to listen carefully to the patient is crucial).

^4^Incidentally, Bion's radical view of the centrality of unconscious intuition for the psychotherapist also seems consistent with the idea advocated here of fostering a receptive attitude that tolerates ambiguity and is open to suspending one's own preconceptions, as IH seems to do, rather than imposing pre-existing assumptions or agendas on the patient and the therapeutic process.
